# Tumor Necrosis Factor and Interleukin-1*β* Modulate Synaptic Plasticity during Neuroinflammation

**DOI:** 10.1155/2018/8430123

**Published:** 2018-05-14

**Authors:** Francesca Romana Rizzo, Alessandra Musella, Francesca De Vito, Diego Fresegna, Silvia Bullitta, Valentina Vanni, Livia Guadalupi, Mario Stampanoni Bassi, Fabio Buttari, Georgia Mandolesi, Diego Centonze, Antonietta Gentile

**Affiliations:** ^1^Synaptic Immunopathology Lab, Department of Systems Medicine, University of Rome Tor Vergata, 00133 Rome, Italy; ^2^Synaptic Immunopathology Lab, IRCCS San Raffaele, Via di Val Cannuta 247, 00166 Rome, Italy; ^3^Unit of Neurology and Unit of Neurorehabilitation, IRCCS Istituto Neurologico Mediterraneo (INM) Neuromed, 86077 Pozzilli, Italy

## Abstract

Cytokines are constitutively released in the healthy brain by resident myeloid cells to keep proper synaptic plasticity, either in the form of Hebbian synaptic plasticity or of homeostatic plasticity. However, when cytokines dramatically increase, establishing a status of neuroinflammation, the synaptic action of such molecules remarkably interferes with brain circuits of learning and cognition and contributes to excitotoxicity and neurodegeneration. Among others, interleukin-1*β* (IL-1*β*) and tumor necrosis factor (TNF) are the best studied proinflammatory cytokines in both physiological and pathological conditions and have been invariably associated with long-term potentiation (LTP) (Hebbian synaptic plasticity) and synaptic scaling (homeostatic plasticity), respectively. Multiple sclerosis (MS) is the prototypical neuroinflammatory disease, in which inflammation triggers excitotoxic mechanisms contributing to neurodegeneration. IL-*β* and TNF are increased in the brain of MS patients and contribute to induce the changes in synaptic plasticity occurring in MS patients and its animal model, the experimental autoimmune encephalomyelitis (EAE). This review will introduce and discuss current evidence of the role of IL-1*β* and TNF in the regulation of synaptic strength at both physiological and pathological levels, in particular speculating on their involvement in the synaptic plasticity changes observed in the EAE brain.

## 1. Introduction

The recognition that soluble mediators of the immune system, namely, cytokines, are constitutively expressed in the central nervous system (CNS) has completely changed our vision of brain functioning [1]. Indeed, the study of the neuroimmune connection is an extraordinary field of research, having strong implications for understanding physiological and pathological conditions [2, 3]. The proinflammatory cytokines IL-1*β* and TNF, released by resident cells of the immune lineage, have been proven to physiologically modulate synaptic plasticity, mainly the Hebbian synaptic plasticity and the synaptic scaling, in different brain areas such as the cortex, striatum, and hippocampus [4, 5].

TNF is a proteolytically cleaved transmembrane protein whose activity is performed through TNF receptor type 1 (TNFR1) and type 2 (TNFR2) [6]. In physiological state, the glial pathway that regulates TNF release is itself controlled by TNF [7], but when the balanced system is strongly disturbed, the homeostatic mechanism fails. This cytokine is an important regulator of synapse function implicated in synaptic transmission and homeostatic synaptic scaling [8, 9].

IL-1*β* is the product of the proteolytic cleavage of its mature form pro-IL-1*β*. IL-1*β* exerts its biological action by binding to IL-1 receptor type 1 (IL-1RI), competing with IL-1 receptor antagonist (IL-1ra), the endogenous inhibitor of IL-1*β* [10]. A bulk of data indicate that IL-1*β* is necessary for synaptic mechanisms, like LTP, underlying learning and memory [4].

When brain levels of cytokines significantly rise as a result of an immune challenge, the scenario about the neuroimmune connection deeply changes. Under this condition, IL1-*β* and TNF, whose basal activity is necessary for maintenance of proper synaptic plasticity, start to exert noxious effects on synaptic transmission. Interestingly, the mechanisms underlying the shift from a healthy immune function to a detrimental one are poorly understood [4]. However, during chronic neuroinflammatory and neurodegenerative diseases, like Alzheimer's disease (AD) and multiple sclerosis (MS), changes in synaptic plasticity due to the effects of these cytokines might also be an adaptive mechanism occurring to compensate for synaptic and/or neuronal loss.

While the physiological regulation of synaptic plasticity by TNF and IL-1*β* has been widely investigated, the involvement of such cytokines in synaptic plasticity alterations associated with neurological disorders is merely speculative and relies only on few studies on animal models. In this respect, due to the recognized pathogenic role of inflammation in MS, many clinical and preclinical studies have been performed to address the role of TNF and IL-1*β* in the modulation of synaptic plasticity [11].

Moving from a brief introduction on the key properties of both synaptic scaling and LTP, the present review summarizes the main evidence for the physiological and pathological functions of IL-1*β* and TNF and their cellular sources in the brain in regulating synaptic plasticity. Moreover, we will discuss data from EAE, animal model of MS, which support a role for both cytokines in synaptic changes and adaptations during neuroinflammation.

## 2. Synaptic Plasticity

Changes in synaptic strength and brain network activity occur either as an adaptive response to environmental stimuli or as a consequence of local insult affecting single or multiple neurons. From development to ageing, several forms of synaptic plasticity coexist and cooperate to maintain proper synaptic transmission and to keep homeostasis in brain circuits. Among others, Hebbian plasticity and synaptic scaling are the most relevant form of synaptic plasticity, whose induction and maintenance underlie not only experience-dependent mechanisms, like memory processes, but also pathological conditions of neuronal perturbations [12]. As reported in the following sections, LTP and synaptic scaling result in the strengthening of the glutamatergic transmission and, although sharing some features, are intrinsically different in nature.

### 2.1. LTP: Properties and Biological Relevance

LTP is a form of synaptic plasticity consisting in long-lasting increase in the synaptic strength between pre- and postsynaptic neurons. It is artificially induced through electrophysiological protocols of high-frequency stimulation [12]. LTP can be experimentally induced in virtually all the excitatory synapses in the brain. However, most of our knowledge about the molecular mechanisms of LTP arises from studies in the cornu ammonis area 1 (CA1) region of the hippocampus, where the main form of LTP is dependent on N-methyl-D-aspartate receptor (NMDAR) activity. Decades of experimental research have led to some key concepts about LTP nature.

Briefly, LTP is (i) cooperative, since it requires the coincident activation of a critical number of synapses; (ii) associative, in a way that weak input, involving a small number of synapses, can be strengthened by the association with a strong input, coming from a larger number of synapses; and (iii) input-specific, because only activated synapses on the postsynaptic neuron are recruited during LTP. This implies that LTP occurs in case of coincidence activity between pre- and postsynaptic neurons in a positive feedback. Indeed, to be triggered, LTP first needs the increased conductance through *α-*amino-3-hydroxy-5-methyl-4-isoxazolepropionic acid receptors (AMPARs), which in turn activate postsynaptic NMDARs (early phase of LTP) [13]. Glutamate massively released from presynaptic terminal binds both AMPARs and NMDARs. However, the latter are activated only once Mg^2+^ is removed from the central pore of NMDAR, and this is achieved by AMPAR-mediated membrane depolarization. Subsequent Ca^2+^ influx through NMDAR channel triggers intracellular signaling cascade necessary for synaptic plasticity [14]. Furthermore, to be persistent over time, LTP requires de novo protein synthesis, necessary for storage of information: the late phase of LTP implies structural changes in postsynaptic density (PSD), which is linked to the induction of immediate early genes (IEG) and the synthesis of proteins like *Arc-Arg*, which stabilizes F-actin filaments and regulate AMPAR membrane expression [15]. On a functional level, compelling studies based on behavioural tests and electrophysiology have clearly linked LTP in the hippocampus with learning [16, 17] and memory [18–20].

Based on subunit composition (NR2A versus NR2B) and localization at synapse (synaptic or extrasynaptic), signaling through NMDAR can induce either neuroprotection [21] or neurotoxicity [22]. Although the causal link between synaptic plasticity and neuroprotection is still not fully elucidated, growing data point to NMDAR-dependent LTP as prosurvival strategy [23], aimed at recovering activity in those neurons, which have lost part of their synaptic inputs.

### 2.2. Il-1*β* Is the Main Immune Trigger of LTP in Physiological Condition

During physiological neuronal activity, several factors have been shown to induce LTP, including the brain-derived neurotrophic factor (BDNF) [24, 25]. However, in the past decades, unexpected interactions between environmental/psychological experiences, immune system, and brain activity have been highlighted, providing evidence for physiological control of learning and memory mediated by the immune system [4]. Research in this field has focused on the effect of cytokines on the induction and maintenance of hippocampal LTP, indicating that IL-1*β*, rather than TNF, is the main immune player in LTP regulation. Indeed, mice with genetic deletion of components of TNF signaling showed unaltered hippocampal LTP [8, 26].

**Figure 1:**
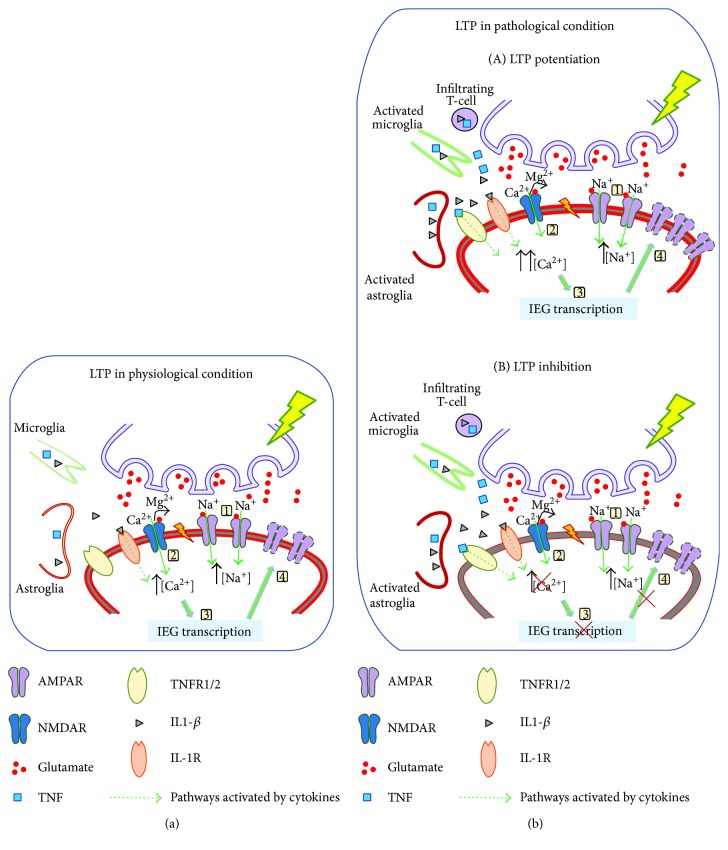
LTP regulation in physiological and pathological states. The triggering of LTP implies coincident pre- and postsynaptic neuron activation. Strong glutamate release from presynaptic terminal promotes membrane depolarization mediated by Na^+^ influx through AMPARs (1), which in turn activates NMDARs by means of Mg^2+^ expulsion from NMDAR pore, thus allowing Ca^2+^ influx (2). Next, the increase of intracellular Ca^2+^ concentration activates a cascade of events involving several molecular players and leads to the induction of IEGs (3), such as Arc/Arg, necessary for structural (increased stability and size of dendritic spines) and functional changes of the PSD and the synthesis and insertion of AMPARs in membrane (4). Physiological levels of IL-1*β* released by both microglia and astroglia contribute to LTP phenomenon (a). During neuroinflammatory disorders (b), activated resident (microglia and astroglia) and infiltrating T-cells strongly release TNF and IL-1*β*, thus generating two possible outcomes of synaptic changes (A, B). As illustrated in the figure, LTP can be either potentiated or prevented through the action of TNF and IL-1*β* interfering with the pathways controlling the molecular and structural synaptic changes occurring during LTP.

After the first observation that LTP induction is physiologically followed by IL-1 gene expression [27], several studies based on genetic knockdown [28] or *in vitro* and *in vivo* pharmacological blockade of IL-1R [29, 30] have indicated the necessary role of IL-1*β* in LTP induction and maintenance. LTP in CA1 region of IL1-R KO mice is absent [28], and intracerebroventricular administration (ICV) of IL-1ra significantly affected both the initial potentiation and the maintenance of LTP [30]. The critical role of IL-1*β* in the maintenance of LTP was demonstrated in *in vitro* experiments: application of IL-1ra 30 min after LTP induction rescued basal synaptic transmission. A putative mechanism by which IL-1*β* modulates LTP involves changes in Ca^2+^ conductance through NMDAR [31]. Noteworthy, *in vivo* manipulations of IL1-*β* signaling were associated with disturbances in memory and learning of mice: compared to wild-type (WT) IL-1R KO mice showed slower rate of learning in the spatial memory paradigm [28], impaired contextual but normal auditory-cued fear conditioning in water *T*-maze paradigm [32]. Moreover, ICV injection of IL-1ra induced similar behavioural phenotype [32]. Overall, these data point to IL-1*β* as the main immune player involved in LTP induction and maintenance as well as memory and learning processes (Figure 1(a)).

### 2.3. Synaptic Scaling: Properties and Biological Relevance

A form of synaptic plasticity, profoundly different from LTP, is the synaptic scaling. The synaptic scaling acts to keep the postsynaptic weights of excitatory synapses around a firing rate set point. Therefore, by definition, synaptic scaling is a homeostatic form of synaptic plasticity, triggered to globally reduce (downscaling) or increase (upscaling) the excitatory drive during chronic inactivity or hyperactivity [33]. Persistent and uncontrolled Hebbian plasticity or reduced number of synapses for pathological reasons can induce synaptic scaling. However, our knowledge on how Hebbian plasticity and synaptic scaling are temporally linked to each other and mechanistically intermingled is still in its infancy [34]. In contrast to LTP, synaptic scaling (i) acts in a negative feedback, (ii) is not input-specific, as it can spread to multiple synapses, and, more interestingly, (iii) mainly relies on AMPAR functioning. Indeed, the excitatory synaptic transmission can be strengthened or weakened by slowly increasing or reducing the number of clustered AMPAR on postsynaptic membrane, respectively [33, 35]. This is a global effect, involving all the synapses of a postsynaptic neuron. As a result of these changes in AMPAR membrane insertion or removal, the conductance of AMPAR is increased or reduced and the PSD area is changed accordingly [36, 37]. Synaptic scaling is associated with the induction or inhibition of Arc/Arg gene [38, 39], leading to increased or reduced rate of surface AMPAR endocytosis with the consequent reduction or enhancement of membrane-expressed AMPARs. Therefore, being involved in both Hebbian plasticity and synaptic scaling, Arc protein seems to play a crucial role in regulating synaptic plasticity [40]. Homeostatic synaptic plasticity has been well documented *in vivo* in visual cortex during experience-deprivation paradigms [41, 42] or in sleep/awake states [31, 43].

### 2.4. TNF Is the Main Immune Trigger of Synaptic Scaling in Physiological Condition

Similar to Hebbian plasticity, synaptic scaling is sensitive to the regulation by molecules of the immune system. If IL-1*β* is definitively associated with constitutive Hebbian plasticity, TNF is invariably associated with synaptic scaling [44, 45]. The first and foremost evidence that TNF is able to alter normal synaptic function was demonstrated in a study where a twofold increase expression of AMPARs on the plasma membrane was detected after an exposure of cultured hippocampal neurons to TNF at different concentrations (0.6–60 nM acute exposure) [9]. Additionally, application of TNFR1 antibody decreased GluR1 surface expression in hippocampal neurons [46], indicating the necessary and constitutive role of TNF in regulating AMPAR membrane insertion and in modifying synaptic strength. Notably, the seminal paper by the Malenka group highlighted the role of glial TNF in inactivity-induced synaptic scaling [8]. Blockade of TNF signaling during prolonged tetrodotoxin (TTX) treatment prevented scaling up of excitatory synapses in hippocampal neurons. Moreover, neurons from TNF KO mice grown on glia from WT mice did show synaptic scaling, while neurons from WT mice grown on glia from TNF KO mice did not [8]. Similar findings were obtained in neurons of the visual cortex [26]. More recently, TNF has been causally involved in size increase of spines close to branches that had recently undergone spine loss [47].

**Figure 2:**
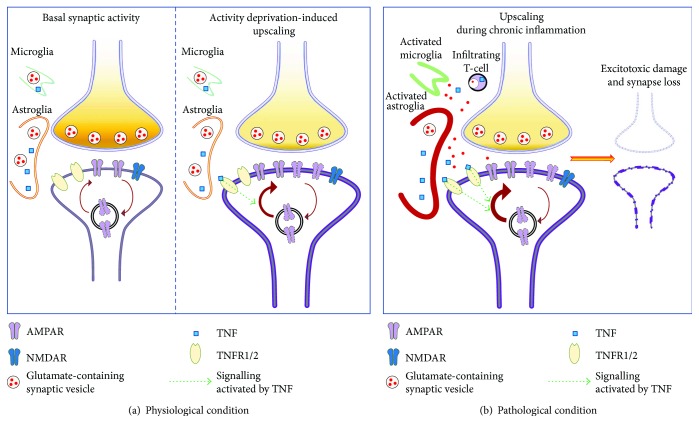
Synaptic upscaling in response to physiological and pathological stimuli. (a) In a physiological state, during basal synaptic activity, AMPARs undergo constant cycles of membrane insertion and removal on postsynaptic neuron. When the synaptic strength driven by the presynaptic terminal is reduced, TNF, released by astroglia, activates a molecular mechanism leading to transient improved insertion of AMPARs on postsynaptic membrane. (b) During acute or chronic neuroinflammation, TNF, massively released by activated microglia and astroglia as well as infiltrating T-cells, indefinitely upregulates the mechanism of membrane AMPAR insertion. In parallel, inflammation affects physiological mechanisms of glutamate clearance at synaptic cleft. This together with enhanced glutamate release from glial cells over activates AMPARs, thus contributing to induce excitotoxic mechanisms and synaptic loss.

Curiously, in contrast to hippocampal and cortical neurons, TNF was shown to downregulate AMPAR membrane expression in striatal neurons, raising the possibility that in this brain region it exerts an adaptive role to limit the strength of synaptic drive from the cortex [48]. Of note, the physiological role of TNF in inducing synaptic scaling has been well documented *in vivo* in the visual cortex of animals subjected to chronic monocular deprivation [26, 47, 49], further supporting the idea that TNF is a critical player in activity-dependent synaptic adaptations (Figure 2(a)).

## 3. Synaptic Plasticity: Synaptic Scaling and LTP during Neuroinflammation

Cells of both the innate (resident microglia and astroglia) and the adaptive (T-cells) immune response have been clearly implicated in the physiological regulation of mood, learning, memory, and experience-dependent synaptic activity [50, 51]. Any changes in brain homeostasis that imply microglia and astroglia activation and/or T-cell infiltration trigger an inflammatory response, which is a mechanism of brain defence and can affect synaptic plasticity. During neuroinflammation, activated microglia, astroglia, and infiltrating lymphocytes specifically interact with neurons and influence their survival either in a positive or in a negative direction depending on the pathologic context, by releasing cytokines [52]. Therefore, we will review current findings about the role of TNF and IL-1*β* in animal models of neuroinflammatory conditions and of neurodegenerative diseases, the latter characterized by chronic inflammation.

### 3.1. Synaptic Plasticity: The Role of TNF during Neuroinflammation

Experimental paradigms of deafferentiation-induced homeostatic plasticity have highlighted that signaling activated by TNF plays a role in the long-term maintenance of synaptic scaling. In hippocampal slices that underwent denervation from entorhinal cortex (EC), glial TNF increased only after 3-4 days postlesion, while the enhancement of excitatory transmission in dentate gyrus (DG) granule cells was observed already after 1-2 days postlesion [53]. Moreover, in the same experimental model of denervation followed by neuroinflammation, it was shown that TNF was involved in LTP maintenance by binding to both TNFR1 and TNFR2 [54].

As already mentioned, TNF exerts different physiological effects in the hippocampus and the striatum [8, 48], and several data suggest that TNF massively released during neuroinflammation may have brain area-specific effects, as well. Accordingly, it has recently been shown that TNF of microglial origin impairs hippocampal LTP in CA1 region, whereas it improves LTP at C-fiber synapses in spinal dorsal horn in a model of peripheral nerve injury, which is associated with memory deficits and pain [55]. Another study on the same model analysed the effect of TNF on hippocampal LTP at CA3-CA1 synapses: LTP was impaired in injured animals and the same effect was observed after intrahippocampal or ICV injection of TNF in healthy mice [56]. These outstanding *in vivo* findings corroborate data from *in vitro* acute application of TNF. It has been shown that TNF impairs dose-dependently LTP induction or maintenance in the hippocampus, by preventing the initial reduction of potentiation (early phase of LTP) and by inhibiting the late increased potentiation (late phase of LTP) [57, 58]. Interestingly, pretreatment of hippocampal slices with TNF after hypoxia improved LTP in the DG [59]. In line with this, by means of transgenic mice overexpressing TNF, other researchers have demonstrated that chronic exposure to TNF potentiates LTP in CA1 region [60] (Figures 1(b) and 2(b)).

### 3.2. Synaptic Plasticity: The Role of IL-1*β* during Neuroinflammation

Several lines of data consistently indicate that increased levels of IL-1*β* inhibit LTP in CA1, CA3, and DG of the hippocampus, either after *in vitro* application of the cytokine or *in vivo* ICV delivery [58, 61–63]. IL-1*β* has been shown to dose-dependently affect Ca^2+^ conductance through NMDARs, being able to improve or inhibit Ca^2+^ influx at low or high concentration, respectively [31]. Moreover, increased brain levels of IL1-*β* may inhibit LTP maintenance by interfering with BDNF signaling cascades, thereby impairing the formation of F-actin in dendritic spines [64]. Among others, these are the putative mechanisms by which IL-1*β* improves or impairs LTP induction.

Regarding *in vivo* studies, stress induced by social isolation and age in rats has been associated with LTP impairment in the DG in correlation with IL-1*β* levels [65]. In animal model of seizure, which is associated with induction of proinflammatory cytokines, hippocampal LTP inhibition and memory deficits were recovered by treatment with anakinra, the human receptor antagonist of IL1-*β*, and not by IL-6 and TNF inhibitors [66]. Likewise, in a model of septic encephalopathy, preincubation of hippocampal slices from septic mice with Il-1ra before the stimulation was found to recover LTP deficiency associated with such pathological condition [67]. Furthermore, in obese mice, intrahippocampal delivery of IL-ra rescued LTP deficiency as well as cognitive impairments at *Y*-maze test [68] (Figure 1(B)).

### 3.3. Synaptic Plasticity: The Role of TNF and IL-1*β* during Neurodegeneration

Increasing interest has been paid to the role of IL-1*β* and TNF during age-related pathological conditions, like AD, since their levels have been found increased in the cerebrospinal fluid (CSF) of these patients [69]. Brain ageing is associated with increased basal levels of cytokines and susceptibility to neuroinflammation, accounting for memory and learning deficits [70]. Of note, neuroinflammation in old people is proposed to contribute to the neurodegenerative cascade typical of AD, namely, *β*-amyloid- (A*β-*) dependent synaptic pathology [71, 72]. Indeed, in line with the aforementioned hypothesis of the inhibitory effect of elevated levels of TNF on LTP in the hippocampus, it has recently shown that in a transgenic mouse model of AD, the peripheral inhibition of the soluble form of TNF attenuates A*β* load, cognitive, and LTP deficits [73]. Regarding Il-1*β* involvement in synaptic pathology associated with AD, IL1-ra treatment partially attenuated A*β*_1–40_ impairment of LTP in the CA1 of hippocampus [30], supporting previous findings suggesting that A*β*_1–40_ induces the release of IL-1*β* [74]. However, in another study, it was proposed that A*β* toxicity was TNF-dependent, since the suppression of LTP induced by A*β* was prevented by pharmacological inhibition of TNF and absent in mice lacking the TNFR1 [75]. These data highlight that cytokines play a crucial role in mediating A*β* synaptotoxicity and mechanisms of memory loss during ageing [72].

Altogether, these findings indicate the detrimental role of high concentrations of TNF and IL-1*β* in both forms of synaptic plasticity during neuroinflammatory and neurodegenerative diseases. It is worth noting that, to date, the occurrence of synaptic scaling in animal models of neurodegenerative diseases has not been addressed. However, it might be hypothesized that in a condition of chronic exposure to high levels of TNF in response to prolonged neuronal activity blockade [8], a kind of super upscaling occurs. This event together with inflammation-impaired mechanisms of glutamate homeostasis regulation subsequently contributes to excitotoxic damage [76, 77] (Figure 2(b)). This issue needs further investigation.

## 4. Evidence of Synaptic Plasticity Perturbations in the Animal Model of MS, EAE

MS is the prototypical neuroinflammatory disorder, initiated by an autoimmune T-cell-mediated reaction against myelin antigen. Demyelination and neurodegeneration are pathological hallmarks of the brains of MS patients and of its animal model EAE [78]. It is worth noting that the synaptic compartment is early perturbed in MS and EAE, and that inflammation is the main trigger of synaptic damage [79]. Such synaptopathy, caused by inflammatory mediators, has been proposed to cogently contribute to cognitive deficits [79], mood disturbances [80], and disability [81] in MS. In particular, cortical Hebbian synaptic plasticity, that is, LTP and LTD, has been explored in MS patients, by means of transcranial magnetic stimulation (TMS) protocols and correlated with the levels of IL-1*β* and TNF [11, 81]. In MS, LTP is favored over LTD and LTP potentiation correlates with IL-1*β* levels in the cerebrospinal fluid (CSF) of MS patients [82]. Moreover, TNF-enriched CSF from MS patients applied to murine brain slices induced the potentiation of glutamatergic transmission, in a way resembling synaptic scaling [83]. Parallel studies on EAE model have confirmed such alterations in basal synaptic transmission and plasticity, providing evidence for a direct involvement of TNF and IL-1*β* in correlation with microglia and astroglia activation and T-cell infiltration [84].

### 4.1. The Role of TNF on Synaptic Activity in EAE

It is properly recognized that in the gray matter of EAE and MS brains, the levels of TNF are severely high [83, 85, 86]. The synaptic activity in EAE mice has been largely investigated by our group. The impact of TNF on synaptic strength has been studied by means of both electrophysiological techniques and biochemical assays. In particular, we observed alterations of frequency and duration of spontaneous and miniature glutamatergic events (sEPSCs, mEPSCs), reporting an increase of both parameters in striatal neurons of EAE mice. Notably, these changes were already evident before the clinical manifestations of the disease [85, 86]. At this stage of the disease, TNF levels have been found increased in EAE striatum [86], raising the possibility that it could be the responsible of such glutamatergic transmission enhancement with an involvement of AMPAR trafficking [8]. Indeed, biochemical assays in synaptosomal preparation of EAE striatum revealed increased expression of GluR1 subunit of AMPAR and its phosphorylation at the Ser845 residue indicative of enhanced AMPAR membrane insertion. Moreover, Arc/Arg mRNA was downregulated in the whole striatum [85]. Together with electrophysiological data, these results are suggestive of synaptic upscaling in the EAE brain [39, 77, 87–89].

The casual link between TNF and enhanced glutamate transmission in EAE striatum was demonstrated by *in vivo* and *in vitro* experiments. Electrophysiological recordings of slices from EAE mice that received ICV treatment with anti-TNF antibody showed the rescue of glutamatergic transmission alteration, while ICV administration of TNF in control mice induced the same enhancement of glutamatergic transmission observed in EAE [86]. Moreover, *in vitro* experiments of long period of incubation (3 h) of control slices with high concentration of TNF (0.6 *μ*M) mimicked the effects of EAE [85]. Such result is apparently in contrast with findings from Lewitus and colleagues (2014), who found that TNF reduced the amplitude of sEPSC and the membrane insertion of AMPAR in the striatum [48]. However, time (1 h) and concentration of TNF (100 ng/ml) in their experimental settings were remarkably different from ours, likely explaining the different *in vitro* results. Of note, the “strong” *in vitro* treatment that we used closely reproduced the EAE glutamatergic transmission potentiation, likely mimicking the effect of chronic exposure of synapses to high levels of TNF. Finally, we confirmed that glial TNF is responsible for the striatal upscaling in EAE: *in vitro* activated microglial cell line applied to control slices increased the duration of glutamatergic spontaneous events and this effect was reversed in the presence of a TNF antibody [85].

The strengthening of glutamatergic transmission in EAE striatum was persistent throughout the disease course. At later stages of the disease, in which inflammation turns into a chronic state, some neurodegenerative features have been described, such as the loss of parvalbumin-positive interneurons (PV+) and of dendritic spines in the gray matter of EAE mice [85, 90], suggesting that inflammatory chronic elevation of TNF may turn physiological upscaling into uncontrolled upscaling, leading to excitotoxic synaptic and neuronal damage [76, 85].

An elegant study published by Habbas and colleagues has demonstrated the involvement of local TNF release in the DG of EAE mice in the strengthening of excitatory transmission in correlation with memory deficits in these mice [91]. The authors found that the excitatory transmission at EC-DG synapses is increased in an astrocytic TNFR1-dependent manner. Indeed, to demonstrate the necessary role of TNF in the potentiation of glutamatergic transmission in circuit involved in contextual learning and memory, they used conditional KO mice for TNFR1 in glial cells. Slices taken from these mice incubated with increasing concentrations of TNF did not show glutamatergic transmission alterations, while the reexpression of TNFR1 in astrocytes rescued the sensitivity to TNF synaptic effect. Moreover, by inducing EAE in this conditional KO mice, they demonstrated that cognitive failure and potentiation of EC-DG glutamatergic transmission are dependent on TNF signaling through astrocytic TNFR1 [91]. Although not fully investigated, along with presynaptic effect of TNF, the authors also found an increase of mEPSC amplitude, consistent with postsynaptic effects of TNF. These results further highlight the role of TNF in synaptic pathology associated with EAE.

### 4.2. The Role of IL1-*β* on Hippocampal Synaptic Plasticity in EAE

IL-1*β* is clearly related to synaptic plasticity rather than upscaling mechanisms in both physiological and pathological conditions (see Sections 2.2 and 3.2). IL-1*β* is essentially involved in the modulation of LTP form of plasticity in EAE mice. In particular, we showed that EAE mice exhibited a favored LTP induction over LTD in the CA1 area of hippocampus. This effect correlated with increased levels of IL-1*β* and was reversed by chronic ICV treatment with IL-1ra [82, 92]. Moreover, preincubation of IL-1*β* on hippocampal slices was able to alter LTP, by inducing a greater potentiation in comparison to control condition and also an inhibition of LTD in CA1 [92]. Of note, any changes in input-output curves as well as in AMPA/NMDA ratio in CA1 were observed in EAE, thus indicating a specific effect on synaptic plasticity induction and maintenance without significant alterations of glutamatergic basal transmission. Based on the above results, we speculated that this effect of EAE on Hebbian forms of plasticity could be the consequence of the reduction of GABAergic inhibition, caused by loss of PV+ GABAergic interneurons [92]. We also demonstrated that *in vitro* activated microglia incubated with control slices inhibited the GABAergic transmission, and that this effect was reversed in the presence of IL1-ra. Considering the role of infiltrating T-lymphocytes in EAE/MS pathology, we tested the hypothesis that these cells, by releasing IL-1*β* [93], might contribute to hippocampal changes in synaptic activity. Experiments carried out with incubation of T-lymphocytes taken from EAE spleen and placed onto hippocampal control slices promoted LTP over LTD, in a way resembling the LTP recorded from EAE slices, and reduced the GABAergic tone [82]. Thus, EAE-specific T-lymphocytes, by suppressing GABAergic transmission in an IL-1*β*-dependent manner, were likely able to lower the threshold of LTP induction. We concluded that IL-1*β* was involved in both the modulation of basal GABAergic synaptic transmission, supposed to precede and contribute to the loss of GABAergic interneurons, and in the potentiation of synaptic plasticity as an adaptive/reparative mechanism.

However, apparently, contrasting data have been reported in literature about hippocampal LTP in EAE [94–96]. Di Filippo and colleagues found that hippocampal LTP is impaired in EAE induced in Biozzi ABH mice, and that IL-1*β* replicates such alteration in *in vitro* experiments. Although not demonstrating a direct link with IL-1*β*, the same authors associated LTP inhibition in EAE to hippocampal-dependent memory defects observed in EAE mice: both behavioural and synaptic alterations in EAE were recovered by suppressing microglia activation by means of peripheral injection of minocycline [95]. Despite the lack of a direct link with IL-1*β*, other studies demonstrated the impairment of hippocampal LTP during the course of EAE [97–99]. In particular, in the paper by Kim et al. (2012), LTP in CA1 region was affected by EAE at both early and late time points and in connection with spatial memory defects [97], while in the investigation by Novkovic et al. (2015), both LTP and cognition were impaired only at late time points [98]. Interestingly, Planche and colleagues correlated impairment of LTP in the DG and of contextual fear memory response in EAE mice with microglia activation, since peripheral administration of minocycline was able to recover both synaptic and behavioural defects [100]. Conversely, Prochnow et al. (2013) investigated presynaptic properties in CA1 hippocampal EAE mice slices reporting a reduction in paired pulse facilitation in comparison with control mice, but no differences were found in LTP induction [96].

As already discussed elsewhere [11, 101], several factors, like EAE model (mice/rats, immunization procedure), different stimulation protocols of LTP, and time points of recordings, which are severely affected by the inflammatory bulk, may explain the contrasting results that have been described in the literature. Even if clear conclusions about synaptic plasticity in CA1 area of EAE hippocampus cannot be drawn, the above data strongly implicate IL-1*β* in synaptic rearrangements during the course of chronic neuroinflammation.

## 5. Conclusions

LTP and synaptic scaling serve as fine-tuning regulators of synaptic strength in the healthy brain and are regulated by IL-1*β* and TNF, which, physiologically act “on demand,” being released in an activity-dependent manner. Interference with these mechanisms can bring to aberrant expression of both forms of synaptic plasticity.

Data discussed in the present review clearly indicate that IL-*β* is largely involved in the constitutive regulation of Hebbian plasticity, while TNF is the main player in homeostatic plasticity. However, such dichotomy is only partially preserved during sustained neuroinflammation. Indeed, although limited, data in literature indicate that in both acute (i.e., ICV injection of cytokine) and chronic (i.e., EAE and AD transgenic model) paradigms of brain inflammation, IL-1*β* is still linked to LTP expression, whereas TNF seems to affect both LTP and synaptic scaling (Figures 1 and 2). To this respect, it should be noted that the biological relevance of an altered expression of synaptic plasticity has been poorly explored in animal models of neurodegenerative diseases, with the exception of MS. In this context, evidence suggestive of an aberrant upscaling mediated by TNF and leading to excitotoxic neurodegeneration has been shown in the striatum of EAE mice. Moreover, TNF-induced glutamatergic transmission enhancement in the DG has been proposed as the synaptic counterpart of cognitive defects in EAE. Regarding Hebbian plasticity, although contrasting, several lines of evidence indicate that LTP expression in EAE is altered in an IL-1*β*-dependent manner. According to these results, aberrant hippocampal synaptic plasticity may contribute either to cognitive impairment or to minimize neuronal and synaptic damage. This issue needs further investigations and may include the effects of other cytokines, like Interleukin-6 (IL-6), and immune molecules, such as major histocompatibility complex type 1 (MHCI), already found to modulate synaptic plasticity [4]. Moreover, the fact that cytokine pathways are highly intermingled, implying mutual regulation lays the ground for a better understanding of the complex interaction between immune system and synaptic activity during the course of chronic neuroinflammation.

## Abbreviations

TNF: Tumor necrosis factor
IL-1*β*: Interleukin-1*β*
LTP: Long-term potentiation
MS: Multiple sclerosis
EAE: Experimental autoimmune encephalomyelitis
CNS: Central nervous system
TNFR1, 2: TNF receptor type 1 and type 2
IL-1RI: IL-1 receptor type 1
IL-1ra: IL-1 receptor antagonist
AD: Alzheimer's disease
CA1, 3: Cornu ammonis area 1, 3
NMDAR: N-Methyl-D-aspartate receptor
AMPAR: *α*-Amino-3-hydroxy-5-methyl-4-isoxazolepropionic acid receptor
PSD: Postsynaptic density
IEG: Immediate early genes
NR2A, B: N-Methyl D-aspartate receptor subtype 2A, B
BDNF: Brain-derived neurotrophic factor
ICV: Intracerebroventricular administration
WT: Wild-type
KO: Knockout
GluR1: AMPA receptor subunit 1
TTX: Tetrodotoxin
EC: Entorhinal cortex
DG: Dentate gyrus
LTD: Long-term depression
TMS: Transcranial magnetic stimulation
CSF: Cerebrospinal fluid
sEPSCs, mEPSCs: Spontaneous and miniature glutamatergic events
PV+: Parvalbumin-positive interneurons
GABA: *γ*-Aminobutyric acid
IL-6: Interleukin-6
MHCI: Major histocompatibility complex type 1.
